# LLM-Mediated Smart Tele-Primary Care for Rural Older Adults: A Caregiver-Centered and Scenario-Assessed Framework for Respiratory Infection Monitoring

**DOI:** 10.3390/s26144610

**Published:** 2026-07-21

**Authors:** Angel Dario Pinto-Mangones, Yair E. Rivera-Julio, Carolina Castellanos-Ramos, Nelson Alexander Pérez-García, Jagger Rivera-Julio, Juan M. Torres-Tovio

**Affiliations:** 1 Systems Engineering Program, Universidad del Sinú, Montería 230001, Colombia; angelpinto@unisinu.edu.co (A.D.P.-M.); juantorrest@unisinu.edu.co (J.M.T.-T.); 2 Department of Computer Science, Corporación Universitaria Americana, Barranquilla 080001, Colombia; yrivera@americana.edu.co; 3 Surgical Instrumentation Program, Universidad del Sinú, Montería 230001, Colombia; 4 Postgraduate Program in Telecommunications, Universidad de Los Andes, Mérida 5101, Venezuela; perezn@ula.ve; 5 Department of Natural and Exact Sciences, Universidad de la Costa, Barranquilla 080002, Colombia; jriveraj@cuc.edu.co

**Keywords:** large language models, tele-primary care, rural health, older adults, caregiver-centered care, respiratory infection monitoring, remote monitoring, clinical decision support, digital health, teletriage

## Abstract

The COVID-19 pandemic accelerated the use of telemedicine and revealed persistent barriers in rural primary healthcare, especially among older adults and caregivers. This study proposes an LLM-mediated smart tele-primary-care framework to support respiratory infection monitoring in underserved communities of Córdoba, Colombia. The framework is based on caregiver-centered teleconsultation, remote monitoring, preventive education, structured symptom reporting, risk-based teletriage, and clinician-supervised digital support. The LLM functions as a controlled conversational interface to collect symptoms, organize patient information, reinforce health education, generate follow-up reminders, and identify predefined warning signs, without replacing clinical judgment or making autonomous diagnostic or treatment decisions. A preliminary scenario-based assessment examined whether the proposed workflow supports coherent triage and appropriate escalation of high-risk cases. Importantly, the described architecture—a telematic system for intelligent-engine access in assisted medicine support (ATIMAMS)—has been implemented and is currently operational at the Systems Engineering Program, Universidad del Sinú (Montería, Colombia), providing intelligent decision support for telemedicine consultations and remote assistance appointments in the study region. Overall, the study presents a context-sensitive, low-barrier, and safety-aware model for strengthening rural primary care, improving continuity of care, and supporting caregiver-mediated respiratory infection monitoring.

## 1. Introduction

The COVID-19 pandemic significantly increased the use of telemedicine as a means to provide continuity of care to patients by reducing the need to travel and have direct contact. For several patient groups this was important. Older adults and those living in rural areas often face issues such as mobility limitation, lack of consistent follow-up, limited access to specialist care and heavy reliance on informal caregiver’s assistance with their daily needs. However, although there has been an increase in use of telemedicine it has shown that access to technology does not equate to continuity of care, compliance with treatment plans or fair outcomes in regard to healthcare, especially in regions with low levels of connectivity, low health literacy and low levels of capacity within the health system [1,2].

Telemedicine may improve access to care, reduce missed appointments and provide continuity of care for populations residing in underserved and Geographically Dispersed Regions. It will be effective if it is adapted to the context, operationally feasible and integrated into primary-care workflows [3,4]. Furthermore, Remote Monitoring Technologies and Digital Care Architectures have provided new avenues for Home-Based Observations, Symptom Tracking, Decentralized Information Exchange and Early Detection of Clinical Deterioration [5,6], which are particularly relevant to acute respiratory infections (ARI) and the identification of warning signs associated with ARI. Early identification and appropriate triage followed by rapid escalation is critical for older adult patients and those with comorbid conditions [7,8].

Beyond general telehealth infrastructure, several AI-enabled systems developed specifically for respiratory-disease diagnosis emerged during and after the COVID-19 pandemic. For example, virtual telemedicine triage tools that combine a conversational AI front end with low-cost vital-sign sensing hardware have been used to differentiate COVID-19 from other common respiratory infections. These systems illustrate that AI-supported respiratory triage is technically feasible, but they typically rely on bespoke hardware integration and disease-specific classifiers rather than the supervised, LLM-mediated conversational layer proposed in the present framework.

Although telemedicine was first proposed as a rapid response to the pandemic, the body of research provides evidence of the lasting utility of telemedicine to support complementary, rather than replacement, in-person healthcare services. Most notably, primary-care service delivery, which includes prevention, early guidance, longitudinal follow-up, and caregiver involvement, is most effectively supported through an in-person encounter [9,10]. Telemedicine supports these efforts by enabling access to clinical expertise for patients living in remote areas or low-resource settings. These areas have multiple barriers to accessing medical services including significant levels of unmet informal caregiving needs [11,12]. Caregivers within these environments perform many roles while caring for their loved ones. These roles can include acting as clinical observers, digital mediators, logistics coordinators, and translating health-related advice into actionable behaviors [3,13]. The successful implementation of any telemedicine model will depend on both clinical accessibility, and the ability of telemedicine models to assist caregivers with recognizing signs/symptoms, accurate reporting of patient data and promoting/engaging in preventable behaviors in the home environment [14,15].

Respiratory infections continue to present one of the largest gaps in terms of declarative knowledge versus sustained behavior. While individuals may have knowledge regarding appropriate actions related to respiratory infection prevention (e.g., washing hands) they are unable to translate this declarative knowledge into consistent action due to factors such as fatigue, social norms influencing their daily routine, lack of correct information, perceived inconvenience associated with engaging in preventative behaviors, low perceived risk of developing respiratory infections and/or lack of trust in health institutions. The “knowledge-behavior” gap is particularly relevant in older adult populations where respiratory infections can develop quickly and caregivers often have to determine when it is necessary to seek additional assistance. As such, primary-care-based telemedicine models need not be limited to synchronous consultation opportunities. Additionally, structured follow-up appointments, personalized education, monitoring activities, triage and feedback mechanisms that promote adherence to established preventive behaviors will likely be needed.

Recent advances in artificial intelligence, particularly large language models (LLMs), provide a new opportunity to strengthen tele-primary-care systems through conversational interfaces capable of collecting symptoms in natural language, generating understandable educational messages, structuring patient-reported information, and supporting clinical workflow documentation [16,17]. Nevertheless, the integration of LLMs into health services requires strict safeguards [18,19]. LLMs may produce inaccurate, incomplete, biased, or overly confident outputs, and they should not be used as autonomous diagnostic systems in vulnerable populations [20,21]. For rural primary care, the appropriate role of an LLM is therefore not to replace clinicians but to operate as a supervised, protocol-constrained, human-in-the-loop support layer that improves communication between caregivers and health teams [22,23].

The present study addresses this need by linking descriptive evidence on prevention-related knowledge, attitudes, and practices among patient–caregiver dyads to the design of an LLM-mediated smart tele-primary-care framework focused on respiratory infection monitoring. Rather than proposing telemedicine as a standalone digital channel, this study conceptualizes tele-primary care as a human-supervised, caregiver-centered, and digitally enabled model that combines synchronous teleconsultation, remote monitoring, preventive education, risk-based teletriage, and an LLM-mediated conversational layer.

The study was guided by three research questions: (i) what prevention-related vulnerabilities are observed among rural patient–caregiver dyads regarding respiratory infection prevention and monitoring? (ii) how can these vulnerabilities be translated into functional requirements for a smart tele-primary-care framework? and (iii) can a scenario-based assessment demonstrate the internal coherence and safety orientation of the proposed teletriage and escalation workflow? Accordingly, the contribution of this study is threefold: first, it characterizes prevention-related behavioral vulnerabilities among rural patient–caregiver dyads; second, it translates these vulnerabilities into functional requirements for an LLM-mediated tele-primary-care framework; and third, it conducts a preliminary scenario-based assessment of the proposed teletriage and warning-sign escalation workflow.

## 2. Materials and Methods

### 2.1. Study Design

A descriptive cross-sectional study was conducted to obtain a contextual snapshot of health-promotion and disease-prevention practices during the COVID-19 period and to identify behavioral patterns relevant to the design of a telemedicine-based primary-care intervention. In addition to the descriptive epidemiological component, the study incorporated an evidence-informed design-science phase oriented toward the development and preliminary scenario-based assessment of an LLM-mediated smart tele-primary-care framework. The study was therefore structured around three linked phases: (i) empirical characterization of patient–caregiver dyads, (ii) translation of empirical findings into functional and safety requirements, and (iii) simulation-based assessment of the proposed teletriage and workflow logic using synthetic respiratory infection scenarios.

The methodological logic combined two complementary domains. This orientation is consistent with recent telehealth research that frames digital care as a sociotechnical intervention requiring alignment between clinical need, user capacity, workflow organization, and technological feasibility [4,24]. First, a clinical and public-health perspective was used to interpret respiratory infection prevention practices, caregiver involvement, and primary-care needs among older adults. Second, a health-systems engineering perspective was used to translate these empirical findings into a digital service architecture integrating data capture mechanism, rule-based teletriage, supervised LLM-mediated interaction, dashboard-based monitoring, and escalation pathways. This dual-domain approach was intended to strengthen both clinical plausibility and technical feasibility [25]. Table 1 summarizes how each methodological stage maps onto a corresponding output for framework development.

### 2.2. Study Context and Sample

The study was conducted within the department of Córdoba, Colombia. Therefore, the targeted population included all patients and their respective caregivers who experienced care needs due to the effects of COVID-19. The analysis unit for this study was therefore defined as the patient–caregiver dyad which reflects the natural interdependencies of managing illness and providing informal support to each other. A dyadic approach is needed when assessing older individuals living in rural environments as these individuals may be dependent upon caregivers not only for physical assistance such as movement and home care, but also digitally related services (e.g., accessing internet, interpreting medical information, contacting healthcare providers).

### 2.3. Sample Selection

Non-probabilistic convenience sampling was employed to select participants. Using a finite population correction method at a 95 percent confidence interval, and an error rate of five percent, the minimal number of dyads necessary to estimate the total population based on the selected sample was determined to be approximately 384 dyads. While convenience sampling has limitations regarding generalizability, the sample size obtained was sufficient to describe behaviors and identify potential gaps in behavior within the target population for the purpose of designing a tele-primary care model.

### 2.4. Data Collection Tool

The data were collected using a 46-item survey tool with three domains:

(i) knowledge of acute respiratory infections;
(ii) beliefs about signs and symptoms of viral;
(iii) preventive actions and health promotion practices.

The survey tool underwent expert review to establish its face validity prior to administration and was pilot tested in a group of twenty participants to verify clarity and comprehensibility, along with cultural relevance. The tool provided an indication of whether participants had declarative knowledge relative to acute respiratory infections, if participants viewed respiratory infection risk as relevant to themselves, and if self-reported behaviors were congruent with recommended prevention behaviors.

### 2.5. Data Analysis

Responses were entered into Microsoft Excel and analyzed in SPSS v26. Descriptive statistics, including frequencies, percentages, means, and standard deviations, were generated. The analysis focused on identifying prevention-related patterns that could inform digital pathway design. Results were interpreted according to three design-relevant dimensions: (i) knowledge sufficiency, (ii) behavioral adherence, and (iii) caregiver-mediated implementation feasibility.

For the assessment-oriented extension of the study, a reproducible Python/Google Colab (2025 runtime version) workflow was developed to generate figures, map empirical findings to design requirements, and test the internal consistency of the proposed teletriage pathway. The computational workflow generated the graphical outputs used in the manuscript, including prevention-practice visualization, the telemedicine pathway, the LLM-mediated architecture, the simulated assessment matrix, performance indicators, and workflow-readiness indicators. This reproducibility layer was added to strengthen transparency and facilitate future prospective validation.

In the design phase, descriptive findings were translated into functional requirements for an LLM-mediated tele-primary-care framework. This process included mapping behavioral vulnerabilities to technological responses, following implementation-oriented approaches that emphasize remote monitoring, mobile access, and progressive integration with existing health-system routines [10,26]. For example, inconsistent adherence to social distancing or home disinfection practices was mapped to personalized educational reinforcement; uncertainty about symptoms was mapped to conversational symptom collection; and delayed help-seeking was mapped to risk-based teletriage and escalation alerts [27].

### 2.6. Framework Development

The smart tele-primary-care framework was developed through an evidence-informed instructional and systems-design process informed by prior work on AI-supported rural care, digital telemedicine infrastructure, and latency-aware IoT architectures [25,28]. The framework retained the original telemedicine components—access and triage, synchronous teleconsultation, remote follow-up, and preventive education—but extended them through a technical layer composed of:

1. a low-barrier caregiver interface, preferably through WhatsApp or a mobile web form;
2. an LLM-mediated conversational assistant for natural-language symptom collection and education;
3. a structured symptom and vital-sign registry;
4. a rule-based respiratory risk stratification engine;
5. a primary-care dashboard for clinician review;
6. an escalation protocol for urgent in-person referral;
7. governance safeguards for privacy, human oversight, and clinical accountability.

The proposed LLM component was deliberately conceptualized as a supervised interface rather than an autonomous clinical decision-maker, in line with recent reviews emphasizing that generative AI in healthcare requires bounded functions, explicit oversight, and prospective validation before clinical deployment [9,23]. Its intended functions are to collect and structure information, communicate validated educational content, detect predefined warning expressions, generate summaries for clinicians, and support follow-up reminders [19,20]. Diagnostic, therapeutic, and referral decisions remain under professional responsibility. The conversational layer is implemented via the OpenAI API using the gpt-3.5-turbo model. Because this is a commercial API, the internal architecture and parameter scale of the underlying model are proprietary and not publicly disclosed by OpenAI; however, the API-level parameters that govern the model’s behavior within this system are configurable and are set as follows for the clinical support use case: a low temperature (0.2) to minimize response variability and prevent speculative outputs, a maximum token limit of 500 per response to enforce brevity and reduce hallucination risk, and a system prompt that explicitly constrains the model to symptom collection, structured reporting, and validated educational content, and that explicitly prohibits autonomous diagnosis, medication recommendation, or contradiction of escalation protocols. End-to-end latency benchmarking (from natural-language caregiver input to structured dataset extraction and dashboard visualization) under real rural network conditions is identified as a required evaluation step of the pilot-implementation phase described in Section 4.4.

### 2.7. Architecture of LLM Layer and Safety Governance

The LLM layer will operate within a constrained, safety-oriented architectural model. All responses from the LLM layer must be based upon locally accepted educational materials, current clinical guidelines, and predetermined escalation procedures. The LLM layer cannot independently provide diagnoses, prescribe medication, alter existing treatment plans or encourage delayed in-person visits when “red flags” have been reported. The safest response to uncertain input is to escalate to a clinician for review instead of providing conversational reassurance.

Functionally, the LLM layer is isolated from the clinical decision-making layer. The LLM layer gathers natural language information, transforms narrative reports from caregivers into a standardized minimum dataset, educates via valid content to prevent disease/condition(s), and generates clinician-focused summary reports. The rule-based teletriage engine determines appropriate action based upon previously defined parameters applied to the standardized data set. Primary-care teams remain accountable for interpreting, prioritizing, and making care decisions related to their patients. Isolation of the two layers was intended to limit automation bias, ensure greater auditability, and allow for integration into primary-care human-in-the-loop workflows.

In addition to operationalizing a safe teletriage framework for rural older-adult care, several other elements must be included to support the safe introduction of the tele-primary-care framework including traceable records of conversations, warnings regarding specific warning signs, clearly stated disclaimers displayed to end-users, data-minimization, role-based access control, end-to-end encryption (E2EE) between the caregiver interface and the clinical dashboard, and regular clinical reviews of generated output. Barriers to understanding and potential over-reliance on automated responses may be exacerbated in rural areas due to low health literacy, digital barriers and caregiver dependency. The present design stage specifies these safeguards conceptually; formal validation of the cryptographic protocol and a security audit of the data pipeline are deferred to the implementation phase.

### 2.8. Design of Scenario-Based Evaluation Framework

Prior to being used with actual patients, the evaluation framework was expanded with a scenario-based evaluation methodology to assess internal consistency, traceability, and orientation toward safety. The scenarios were created as synthetic but clinically relevant representations of surveillance for respiratory infections in rural older adults. Each of the scenarios represented different levels of risk and included varied combinations of incomplete home measurement data, caregiver uncertainty, comorbid conditions, transportation barriers, digital barriers and uncharacteristic deterioration patterns in order to account for prior evidence of symptom co-occurrence and exploratory risk diagnostics using AI [22,29]. Concretely, the sixty scenarios were organized into three illustrative severity bands of twenty cases each (low-, moderate-, and high-severity construction), and within each band the minimum-dataset fields defined in Table 2 (age, SpO_2_, dyspnoea severity, confusion, chest pain, fever duration, comorbidity, caregiver-concern score, respiratory rate, and transport barrier) were varied by simple deterministic cycling formulas indexed by case number, rather than by a stochastic generative model; this combinatorial construction, fully specified in the accompanying code, is what is meant by “synthetic” throughout this manuscript. One in every six cases in each band was constructed with an unavailable SpO_2_ reading, representing the realistic unavailability of a pulse oximeter in some rural households.

Both the reference triage label and the workflow-output label for every scenario are computed by the same deterministic rule function (the numerical thresholds in Table 3), so that no label is manually assigned. The reference label is computed from each scenario’s complete underlying values, representing the triage decision a clinician would reach with full information. The workflow label is computed from the data as it would actually be available to the LLM-mediated workflow, i.e., after the simulated unavailability of the SpO_2_ reading described above; when this reading is missing in a patient who is already vulnerable (comorbidity present, caregiver-concern score at or above the moderate threshold, or age ≥75 years), the workflow conservatively escalates the case by one risk tier rather than assuming the missing value is benign. This process did not evaluate an actually implemented LLM with real users and therefore does not support claims of effectiveness as a clinical tool. Instead, the process evaluated whether the framework’s rule-based logic behaves safely and consistently when fed structured, synthetic data that includes realistic measurement gaps.

Evaluation focused on evaluating critical-safety outcome metrics (consistent with safety oriented AI evaluation methodologies and respiratory symptom analytics): agreement with reference triage labels, sensitivity for identifying high-risk escalations, specificity for preventing unnecessary high-risk classifications, balanced accuracy across risk levels, complete capture of the minimum dataset, and usability of actions initiated by dashboard display. Summary statistics were derived using a confusion matrix and Cohen’s kappa statistic [30,31]. Early stage evaluation methodologies for AI-enabled decision-support systems emphasize transparency, human oversight, safety testing and impact on workflow rather than autonomous diagnostic performance [32,33].

### 2.9. Ethics

This study complied with national ethics guidelines for research involving human subjects. Study participation was strictly voluntary; informed consent was collected from all study participants; and participant confidentiality was maintained throughout the study. As the study involved both survey-based characterization and development of a care pathway there was no clinical intervention component of the study protocol. Institutional review and informed-consent procedures specific to this study are reported in the Institutional Review Board Statement and Informed Consent Statement at the end of this article.

There are additional ethical implications for implementing an LLM-mediated telehealthcare platform that need to be addressed prior to rollout. Examples include obtaining explicit informed consent for digital interaction, minimizing data collection, ensuring safe storage of sensitive healthcare information, educating users that they should continue to rely on healthcare professionals for diagnosis/treatment and not on automated conversational output, documenting audit trails of generated output, establishing escalation rules for urgent symptoms, and mitigating against inherent biases within algorithms. Transparency and human oversight are essential in protecting against automation bias, false reassurance and/or overreliance on conversational AI in vulnerable populations such as older adults and rural caregivers.

## 3. Results

There are four interconnected areas where the empirical data will be organized. These include the empirical characterization of preventive practices among the dyad sample members; the integration of those empirical results into a telemedicine-based care path for primary healthcare services; the expansion of that care path to create an artificial intelligence-based smart tele-primary-care service; and a scenario-based evaluation of both the teletriage and workflow methodology.

### 3.1. Preventive Practice Findings

Descriptive statistical analysis indicated that participants had good declarative knowledge of COVID-19-related topics; however, this did not result in consistent practice regarding prevention. It is critical to note that if a digital health intervention focused solely on disseminating information it would not meet the behavioral and operational vulnerabilities previously identified within the population.

Nearly 90 percent of respondents indicated they had sufficient knowledge about symptoms, how the disease is transmitted and what preventative steps can be taken. However, a smaller number (approximately 1.6 to 2.9 percent) reported that they did not have a full understanding of these concepts, resulting in potential gaps in knowledge that could lead to delayed identification of warning signs in older adult populations.

Compared to other prevention practices, diet-related behaviors appeared favorable with approximately 80.23 percent of the respondents indicating they prepared all meals at home and approximately 19.74 percent continued to eat outside of their home. Conversely, biosecurity behavior was highly variable. While agreement was moderate to strong for certain behaviors (i.e., wearing masks—60.18 percent and disinfecting floor surfaces and bathroom fixtures—48.14 percent), agreement was less robust for behaviors including disinfesting shoes prior to entering their homes (rejected by approximately 59.47 percent of respondents) and disinfecting keys and door handles (agreement was obtained from only 27.98 percent).

Moreover, greater than half (50.44 percent) of respondents acknowledged that their current practices either violate social distancing guidelines or avoid physical greeting when experiencing upper respiratory symptoms, indicating a large degree of behavioral vulnerability to household and community wide spread of the virus. Additionally, nearly one-fifth (21.57 percent) chose not to answer questions regarding limiting trips away from home to essential events, which may indicate ambivalence, fatigue or social desirability biases toward restrictions.

**Figure 1:**
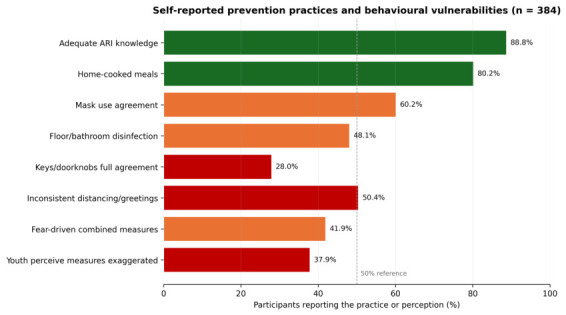
Self-reported prevention practices among study participants (*n* = 384).

Motivation to engage in preventive behaviors was also heterogeneous. Nearly 42 percent—particularly among older adults—reported engaging in multiple preventive behaviors due to fear of contracting the virus, while nearly 38 percent believed that younger individuals view COVID-19 protective measures as excessive. Collectively, these results illustrate a significant dichotomy between awareness of COVID-19 and sustained adherence to preventable behaviors, thereby emphasizing the need for interventions that transcend mere dissemination of information to provide culturally sensitive support, facilitate caregiver involvement and provide individualized encouragement. Figure 1 summarizes these self-reported prevention practices across the study sample.

### 3.2. Analysis-Based Interpretation of Behavioral Vulnerabilities

Three empirically derived design vulnerabilities were observed. First, the high level of knowledge among participants, coupled with variability in preventive behaviors illustrates that a single major barrier to implementation is primarily behavioral in nature rather than informational. Secondly, the fact that there exists a dyadic unit consisting of an older adult and caregiver indicates that success will depend on the caregiver’s ability to identify symptoms, effectively communicate with medical professionals and maintain adherence to recommendations in the home environment. Finally, the heterogeneity of preventive motivation underscores the necessity for continuous communication and personalized reinforcement rather than relying on static educational communications. Table 4 translates each empirical result into its corresponding clinical interpretation and digital design requirement.

### 3.3. Telemedicine Evidence-Based Care Pathway

The empirical findings influenced the creation of an integrated telehealth pathway specifically developed for elderly patients and their caregivers residing in rural and low-income communities. The proposed pathway utilizes synchronous teleconsultation along with remote monitoring capabilities to improve prevention, initial education and continuity of follow-up for acute respiratory infections in primary-care settings.

Rather than replacing emergency or face-to-face care, the pathway was conceived as a complementary mechanism to expand reach and improve continuity. Its design reflects three practical priorities emerging from the descriptive results: first, the need to bridge the gap between knowledge and preventive behavior; second, the importance of supporting caregivers as active participants in home-based care; and third, the necessity of adapting communication and follow-up strategies to local access and learning conditions.

**Figure 2:**
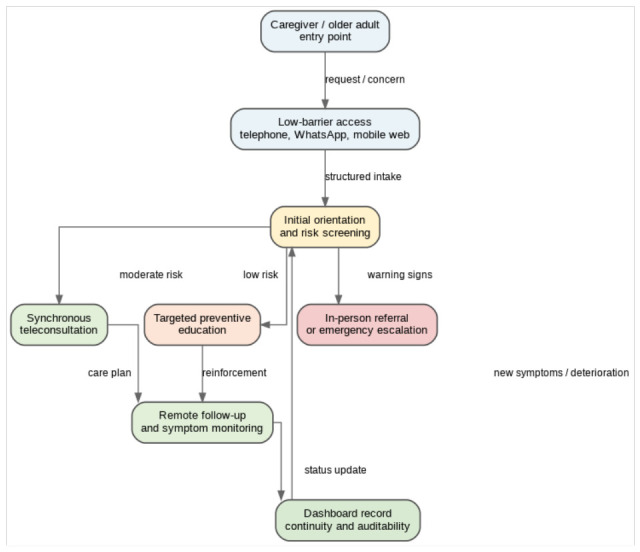
Comprehensive access pathway for primary-care telemedicine.

In functional terms, the pathway includes four core components: access and triage, synchronous teleconsultation, remote follow-up, and targeted preventive education. These components, summarized in Table 5 and illustrated in Figure 2, are coordinated to support both clinical guidance and behavior-oriented reinforcement.

### 3.4. LLM-Mediated Smart Tele-Primary-Care Architecture

**Figure 3:**
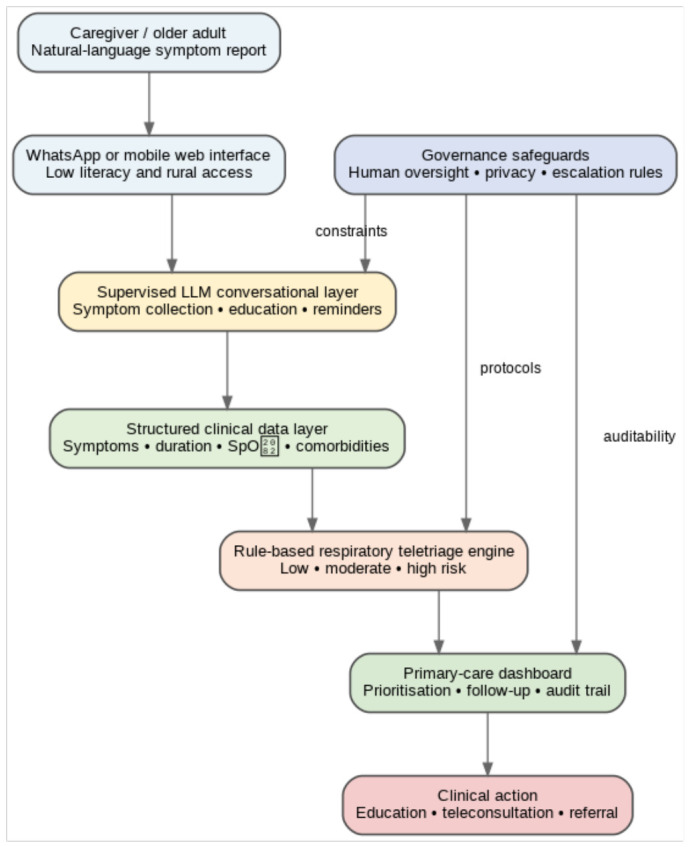
LLM-mediated smart tele-primary-care architecture for rural respiratory infection monitoring.

The original pathway was extended into a smart tele-primary-care architecture in which the LLM functions as a supervised conversational layer between the patient–caregiver dyad and the primary-care team. The architecture is not designed to automate medical diagnosis. Instead, it is designed to improve communication, structure symptom data, detect predefined warning signs, reinforce preventive education, and reduce the documentation burden for clinical teams. Figure 3 depicts the resulting six-layer architecture.

The proposed architecture contains six layers. Its layered structure was designed to separate communication, data structuring, clinical rules, and professional judgment, a separation that is recommended in AI-enabled healthcare systems to reduce automation bias and improve auditability [20,21]. The first layer is the patient–caregiver interaction layer, which prioritizes low-barrier channels such as WhatsApp or mobile web forms. The second layer is the LLM conversational layer, which translates caregiver language into structured information. The third layer is the clinical data layer, where symptoms, vital signs, comorbidities, and duration are represented in a standardized format. The fourth layer is the rule-based respiratory teletriage engine. The fifth layer is the clinical dashboard, where nurses or physicians review cases and make decisions. The sixth layer is the care action layer, which may include education, teleconsultation, follow-up, or referral to in-person care. Table 6 details the function, input, output, and safeguard associated with each LLM component.

### 3.5. Clinical Data Model for Respiratory Infection Monitoring

The minimum clinical dataset required by the framework is to be captured feasibly in rural locations and to be useful as a basis for primary-care triage. This focus on capturing clinically useful data (and in this case, a “minimum” dataset) has parallels in both remote patient monitoring and wearable health literature where there is an emphasis on remote data being actionable, understandable and not relying too heavily upon sophisticated infrastructure [11,12]. A “minimum” dataset would therefore include those variables from which reliable caregiver reports could be generated and those from which rapid clinician interpretation could take place. It will consist of demographic risk factors, symptoms, vital signs, comorbidities, functional deterioration and contextual barriers [27], as detailed in Table 2.

### 3.6. Risk-Based Teletriage Logic

The proposed teletriage engine classifies cases into three operational categories, reflecting prior telehealth literature that emphasizes structured triage, timely escalation, and hybrid continuity between remote and in-person care [24,26]. The categories are low risk, moderate risk, and high risk. This classification is not generated autonomously by the LLM. Rather, the LLM structures the information, and the rule-based teletriage engine applies predefined clinical criteria. The final decision remains under the responsibility of the primary-care team. Table 3 presents the indicative criteria and recommended action associated with each risk level.

The teletriage logic should be adapted to local clinical protocols, available resources, and national health regulations. Importantly, the model should be conservative in older adults, because clinical deterioration may be atypical and because delayed referral may increase risk. For this reason, caregiver concern should be treated as a clinically meaningful signal rather than a subjective inconvenience: a caregiver-concern score at or above the moderate threshold is, by itself, sufficient to trigger at least a moderate-risk classification. As an additional conservative safeguard, when the pulse-oximetry reading is unavailable (e.g., no device at home) in a patient who is already vulnerable—defined here as presenting a comorbidity, a caregiver-concern score at or above the moderate threshold, or age ≥75 years—the workflow escalates the case by one risk tier rather than assuming the missing reading is benign.

### 3.7. Dashboard and Workflow Indicators

The operational interface for the healthcare team is the dashboard. Unlike other AI outputs, it will serve as a workflow-support tool (as opposed to a standalone output), as recommended by remote monitoring research that emphasize longitudinal tracking, clinical interpretation and team review [11,27]. The main role of the dashboard is to take caregiver-reported data and turn it into clinical actionable workflow data. Thus, it should prioritize both clinical relevance and simplicity, avoiding unnecessary technological sophistication. Table 7 lists the indicators proposed for this dashboard.

### 3.8. Implementation Safety Protocols

In order to responsibly deploy LLMs within tele-primary care environments, there must be protections for safety at multiple levels (clinical, technical, ethical, organizational). A number of recent reviews of LLMs in healthcare concur that all applications of LLMs must have strictly defined use cases, verified source knowledge, human supervision, and well-defined limits to their ability to produce harm prior to deployment to vulnerable populations [19,21]. For the described system, the LLM is to be limited by validated script sets; the LLM is to retrieve relevant educational materials from an approved repository; the LLM is to escalate as per rule-based criteria; and the LLM is to be reviewed by clinicians. The LLM should never autonomously diagnose patients, recommend medications, contradict emergency protocols, or advise against seeking face-to-face medical attention when warning signs exist [22]. Table 8 maps each identified risk to its potential consequence and mitigation strategy.

### 3.9. Assessment of Teletriage Workflow via Scenario-Based Evaluation

The purpose of this evaluation was to assess whether the proposed framework can convert caregiver-reported information into primary-care workflow categories which are actionable and yet remain within a conservative risk model. This synthetic assessment included sixty different respiratory infection scenarios divided among three different profiles (low risk, moderate risk, and high risk) with respect to the degree of risk associated with each. Each respiratory infection scenario was developed using evidence that combinations and co-occurrences of respiratory symptom patterns may contain clinically useful information regarding the stratification of risk [29]. The scenarios intentionally included cases with incomplete home measurements, rural transport barriers, caregiver uncertainty, comorbidities, and atypical deterioration patterns in older adults. As described in Section 2.8, the rule-based engine itself was implemented as deterministic threshold logic within the Python/Colab workflow, applied to the structured fields of the synthetic minimum dataset rather than to a deployed conversational LLM.

**Figure 4:**
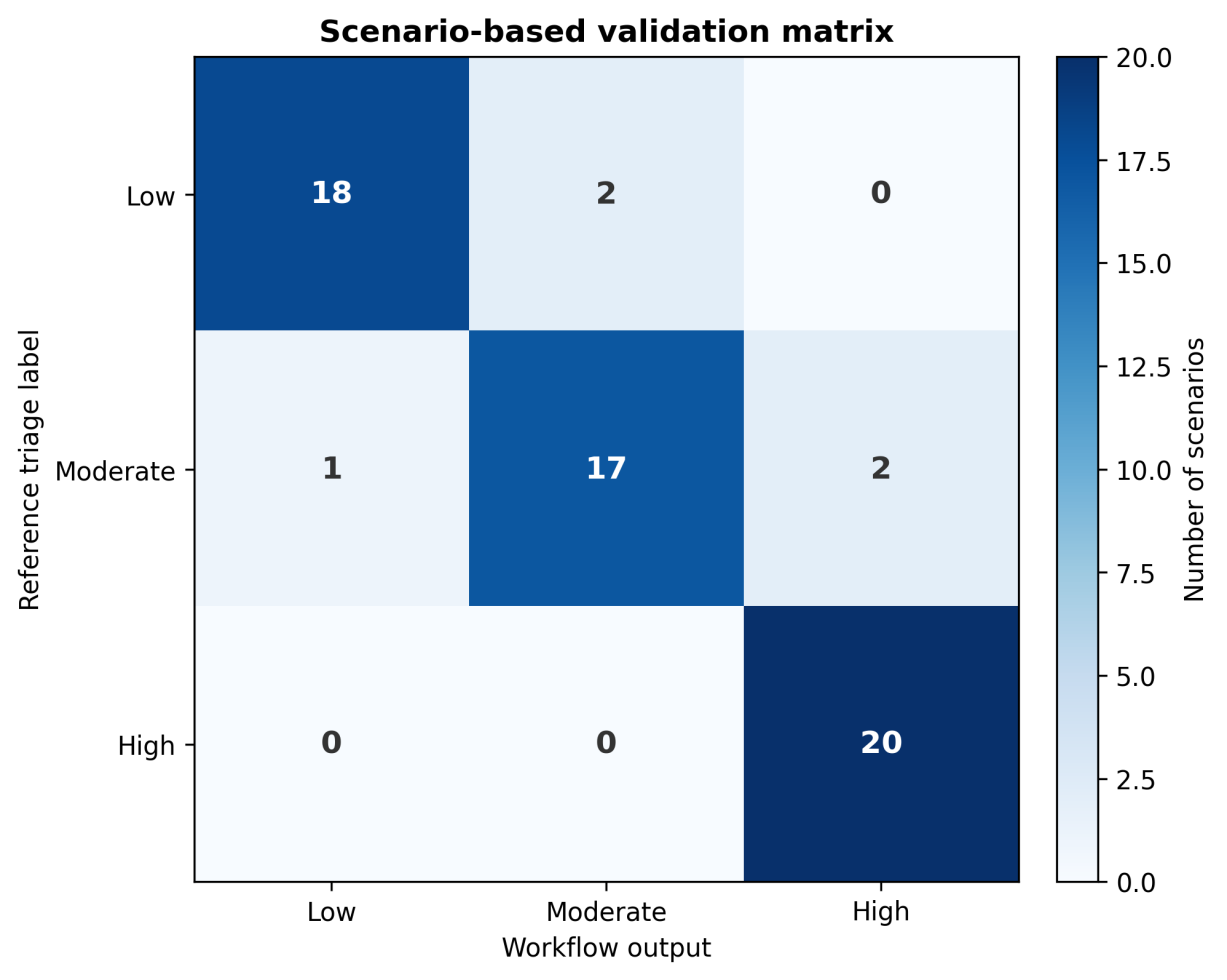
Scenario-based assessment matrix for the proposed respiratory teletriage workflow.

The simulated workflow showed high overall agreement with the reference triage labels within this preliminary assessment. All four classification discrepancies were escalations rather than de-escalations: one case constructed as low risk was routed as moderate risk, and three cases constructed as moderate risk were routed as high risk, in every instance because a pulse-oximetry reading was unavailable in a patient who was already vulnerable (comorbidity, elevated caregiver concern, or age ≥75 years), triggering the conservative missing-data escalation rule described in Section 3.6. No case was ever de-escalated, and no high-risk warning-sign case was routed as low or moderate risk in the simulated assessment set. This behavior is important because the framework is intended for vulnerable older adults, where conservative escalation is safer than under-triage. Figure 4 presents the resulting assessment matrix.

**Figure 5:**
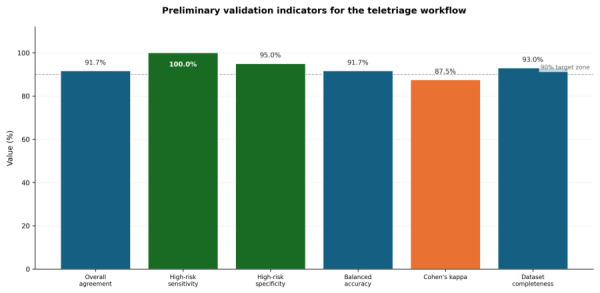
Preliminary performance indicators from the scenario-based assessment exercise.

The performance indicators suggested that the proposed workflow logic was internally coherent for preliminary assessment purposes. The highest priority indicator was sensitivity for high-risk escalation, because false reassurance in older adults with respiratory deterioration would represent the most clinically relevant safety failure. The simulated workflow correctly escalated all predefined high-risk cases, while maintaining acceptable specificity for avoiding unnecessary high-risk classification. These indicators are summarised in Table 9 and Figure 5.

**Figure 6:**
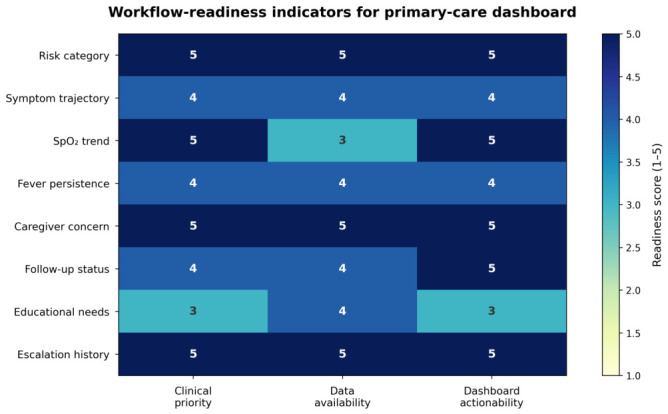
Indicators for workflow readiness as derived from the simulated respiratory monitoring scenarios.

Beyond the traditional measures of classifier performance, the case study evaluated whether the designed architecture was ready to support its intended workflow. Indicators on the dashboard were organized into seven categories including clinical priority, symptom trajectory, SpO_2_ trends, concerns expressed by caregivers, follow-up status, educational needs, and escalation history. Together these categories provide a means to track the activity associated with each patient case allowing the primary-care team to quickly determine which patients are due for immediate review, scheduled teleconsultations or normal preventative reinforcement, as illustrated in Figure 6.

Thus, while the use of the “scenarios” as part of the assessment process should be viewed as a form of internal testing (as opposed to external testing), in terms of demonstrating that the proposed architecture is logically coherent, provides measurable indications of system operation, and produces a computationally reproducible workflow, it represents a valuable extension of the manuscript’s conceptual framework and establishes a clear pathway to pilot implementations, subsequent evaluations and eventually comparative studies against other telemedicine workflows.

## 4. Discussion

In conjunction with behavioral research concerning how a particular group of people (vulnerable rural elderly) behave regarding their health and well-being in relation to digital technologies, this study adds to the body of research concerning telemedicine and digital health through providing a structural representation of a logical architecture for an LLM-mediated smart tele-primary-care system. Additionally, this study is consistent with recently published research articles that describe telemedicine as a long-term method of delivering healthcare to older adults, caregivers and underserved communities, as opposed to just being used during public health emergencies (i.e., pandemics). The major finding of this study relates to the fact that individuals’ level of knowledge regarding their own health does not necessarily translate into their consistently practicing good health prevention behaviors. This gap is especially pronounced in rural areas with many caregivers and is influenced by their daily activities, culture, logistics issues and availability of technology.

One of the significant contributions made to existing conceptual frameworks for telemedicine is the ability to assess the developed architecture using a scenario-based evaluation process. While this process will never take the place of prospective clinical trials/evaluations, it will show that the architecture can be implemented, tested and audited. For researchers conducting Level Q1-Q2 digital health research, one of the biggest challenges facing them today is developing AI-enabled architectures that have measurable performance metrics, safety logic, workflow compatibility and some demonstrable path forward for implementing these solutions in practice, versus simply presenting an architectural description.

Therefore, based upon the findings of this study there appears to be sufficient rationale for transitioning from informationally based models of telemedicine towards smart tele-primary-care models that are focused on both the individual and their caregivers. The disparities in distancing behavior, domestic biosafety procedures and perceived risk demonstrated in this study indicate that the problem faced by individuals in maintaining healthy practices does not appear to be solely related to lack of education. Instead, it would seem that problems exist in regard to adherence to new practices, practicality, social acceptance and continued reinforcement. This conclusion is consistent with previously published systematic reviews that indicated telehealth is generally effective when it provides continuity, accessibility, follow-up and adaptability in meeting the needs of older adults and vulnerable populations.

For clinicians, the proposed architecture is relevant as it integrates respiratory infection monitoring with what occurs in reality when caring for older adults. Older adults may exhibit non-traditional signs of illness, can rapidly deteriorate and rely on caregivers to recognize and communicate symptoms with service providers. Therefore, the caregiver is not secondary but primary in regard to operating tele-primary care. By placing the caregiver at the beginning of the digital pathway, the framework recognizes the actual organization of home-based care in rural environments.

From an engineering perspective, this framework contributes an architecture that isolates conversational intelligence from clinical decision-making. Isolating conversational intelligence from clinical decision-making is especially relevant since LLMs can assist with documentation, summarization and conversational data capture; however, LLMs can still experience errors, over-confidence and contextual loss. This isolation is necessary. LLMs organize information and enhance communication while rule-based protocols and human clinicians continue to be responsible for determining triage, diagnoses and treatment decisions. Modularity decreases risks, increases auditability and allows the architecture to better interface with real-world primary-care systems. Also, modularity allows incremental implementation: health organizations can start by collecting symptoms and sending educational messaging, next by adding dashboard monitors, and followed by incorporating more sophisticated analytics once validated.

The proposed model also addresses a frequent weakness of telemedicine implementations: lack of integration into workflow. A video or telephone consultation alone does not ensure continuity. In contrast, the proposed model includes follow-up status, risk categories, caregiver concern, educational needs, and escalation history. These elements create a feedback loop that allows primary-care teams to prioritize patients, detect missed follow-ups, and evaluate the quality of remote care.

The LLM layer is especially valuable in the communication gap between rural caregivers and clinical teams. It also creates a bridge between natural-language reporting and structured clinical workflows, a use case consistent with emerging applications of LLMs for health-data organization and decision-support augmentation. Caregivers may describe deterioration using non-technical language, such as “breathing strangely”, “not looking well”, or “becoming very weak”. A supervised LLM can help structure these descriptions into clinically relevant categories for review. However, because such interpretation is probabilistic and may be wrong, it must always be embedded within human oversight and conservative escalation criteria.

The model is also aligned with the transition from pandemic telemedicine to sustainable digital primary care, particularly in Latin American and rural contexts where digital inclusion, workforce readiness, and low-barrier access remain central implementation constraints. During the pandemic, telemedicine was often used as an emergency substitute for in-person consultation. In the post-pandemic context, its value depends on whether it can become an organized, equitable, safe, and scalable extension of primary care. The proposed framework advances this transition by integrating prevention, monitoring, education, teletriage, caregiver support, and clinical workflow optimization.

### 4.1. Clinical Implications

The clinical implications are fourfold. First, respiratory infection monitoring in older adults should include caregivers as active participants rather than passive companions. Second, telemedicine should incorporate structured symptom follow-up rather than isolated consultation events. Third, warning-sign detection should be conservative, especially when remote data are incomplete. Fourth, education should be repeated and adapted to the caregiver’s context, because knowledge alone may not produce adherence.

### 4.2. Technical Implications

The technical implications are equally important. First, the system should be designed as a layered architecture separating user interaction, data structuring, risk stratification, dashboard visualisation, and clinical action. Second, the LLM should be restricted to support functions rather than autonomous clinical decisions. Third, the minimum dataset should be feasible for rural caregivers and should not depend exclusively on advanced devices. Fourth, the dashboard should privilege actionable indicators rather than excessive data volume. Fifth, the system should maintain audit trails to support accountability and quality improvement.

### 4.3. Public Health and Policy Implications

At the public-health level, the proposed model can support health institutions seeking to expand reach without weakening clinical accountability. For rural regions, LLM-mediated tele-primary care could reduce avoidable travel, improve early recognition of deterioration, support caregivers, and strengthen continuity between households and primary-care teams. At the policy level, implementation would require guidance on data protection, professional responsibility, AI governance, digital inclusion, and integration with existing health information systems.

### 4.4. Implementation Readiness

The framework described in this manuscript has progressed beyond the design stage: the telematic architecture for intelligent-engine access in assisted medicine support (ATIMAMS) is currently implemented and operational at the Systems Engineering Program, Universidad del Sinú (Montería, Colombia), providing intelligent decision support for telemedicine consultations and remote assistance appointments in the study region. The conversational layer is implemented using the gpt-3.5-turbo model via the OpenAI API with the parameter settings described in Section 2.6. The present article constitutes the formal documentation and scientific framing of this deployed system.

The following staged validation activities are now actively underway or planned as the next formal research phase: (i) systematic usability evaluation with caregivers and primary-care professionals; (ii) independent validation of educational content; (iii) prospective assessment of clinical outcomes, such as time to consultation, escalation accuracy, caregiver adherence, and burden; (iv) hardware-level sensor integration and end-to-end latency benchmarking under real rural network conditions; and (v) comparative assessment against standard telemedicine workflows. These activities will build on the operational baseline the system now provides.

### 4.5. Limitations

This study has several limitations. First, its cross-sectional descriptive design does not allow causal inference. Second, the use of convenience sampling limits representativeness and external validity. Third, the findings are based on self-reported practices and may therefore be affected by recall bias or social desirability bias. Fourth, the scenario-based assessment used synthetic cases and should therefore be interpreted as an internal coherence and safety test rather than as evidence of clinical effectiveness. Fifth, although the described system is currently operational and providing support for telemedicine consultations and remote appointments, the formal prospective evaluation of real-world diagnostic accuracy, outcome improvement, user adherence, and cost-effectiveness has not yet been completed; the present article documents the architecture, its empirical grounding, and its preliminary scenario-based coherence assessment, and formal clinical outcome data are the subject of ongoing and planned follow-up research. Sixth, while the system is currently operating in the study region and therefore is being tested under real rural connectivity constraints and clinician workload conditions, systematic evaluation of institutional governance compliance, connectivity-loss handling, and network-latency impact on triage performance has not yet been formally reported and constitutes a planned component of the next research phase. Seventh, the framework does not yet incorporate device-level sensor characteristics: unlike hardware-integrated telemedicine triage tools that combine wearable or bedside sensors with an AI front end, the present scenario-based assessment used idealized symptom and vital-sign values and did not model motion artifacts, sensor misalignment, packet loss, or other sources of real-world signal noise; incorporating such physical-layer constraints is identified as necessary future work rather than a claim of the current study.

These limitations are important, but they do not diminish the study’s value as a contextually grounded design contribution. Rather, they define the next research step: implementation and evaluation under real-world conditions. Future studies should include prospective pilots, mixed-methods acceptability assessment, clinical safety evaluation, economic analysis, and comparison with standard telemedicine workflows.

### 4.6. Conclusions

This study provides strong evidence that when appropriately designed, such as an integrated and context-specific pathway, telemedicine is a viable alternative (or complement) to strengthen primary care in rural and underserved communities. Telemedicine has a number of advantages over traditional forms of delivering primary care including increasing access, supporting continuity of care, promoting prevention, and providing the opportunity for caregivers to become involved in the care process.

Through combining 384 patient–caregiver dyad case studies using both an empirically supported design methodology and scenario-based assessments, this study provided a new LLM-mediated smart tele-primary-care framework that integrates tele-consultations, remote monitoring, preventative education, risk-based teletriage, caregiver support and clinician-supervised follow-up. The emphasis of the proposed framework was placed upon behavioral reinforcement, human oversight, caregiver involvement and adaption to local social and cultural contexts.

These results clearly indicate a large disparity exists between what individuals know about their health and their ability to sustain healthy behaviors, thus, future telehealth initiatives will need to shift away from simply disseminating information and toward ensuring compliance, establishing feasibility within the patients’ environment, conducting longitudinal follow-up and integrating tele-health into workflows. Therefore, telemedicine should be viewed as an organizational, relational and technology-based extension of primary care rather than simply a replacement for face-to-face consultations.

As an additional novel but careful contribution the proposed LLM layer enables supervised conversational support as opposed to autonomous clinical decision-making and therefore should be governed by regulations similar to those established for Clinical AI and LLM-based healthcare systems. Specifically, the proposed LLM layer could provide: natural language symptom collection; educational reinforcement; caregiver guidance; clinical summarization; and follow-up reminder capabilities; however, it cannot function as an independent diagnostic or treatment agent. The appropriate utilization of the proposed LLM layer is dependent upon predefined protocols, validated content, data governance, auditability, and human-in-the-loop clinical supervision.

Practically speaking, the proposed framework presents a scalable model for healthcare organizations that seek to increase service reach/efficiency while decreasing caregiver burdens, particularly so if low-tech access routes, remote monitoring, and hybrid continuity pathways are maintained. Crucially, the telematic architecture described in this article is not a prospective proposal: it has been implemented and is currently operational at the Systems Engineering Program, Universidad del Sinú (Montería, Colombia), providing intelligent decision support for telemedicine consultations and remote assistance appointments in the study region. The present article constitutes the formal scientific documentation of this deployed system. Ongoing evaluations of effectiveness, safety monitoring, cost/benefit analyses, and policy translations will determine the full extent to which the operational framework delivers measurable improvements in rural primary-care outcomes.

## Figures and Tables

**Table 1:** Methodological sequence linking empirical evidence, framework design, and preliminary assessment.

Stage	Purpose	Output for Framework Development
Empirical characterization	Identify knowledge, attitudes, and preventive practices among patient–caregiver dyads	Behavioral vulnerabilities and contextual barriers relevant to rural respiratory infection monitoring
Functional translation	Convert empirical findings into design requirements	Caregiver-centered requirements for education, symptom reporting, follow-up, and escalation
Framework design	Structure the tele-primary-care architecture	Integrated model combining teleconsultation, remote monitoring, LLM-mediated interaction, rule-based teletriage, and dashboard review
Scenario-based assessment	Examine internal coherence before deployment	Preliminary evidence of workflow traceability, warning-sign escalation, and implementation readiness

**Table 2:** Dataset for LLM-mediated respiratory infection monitoring.

Data Domain	Variables	Clinical Relevance
Demographic and risk profile	Age, sex, rural location, living situation, caregiver availability	Identifies vulnerability and care dependency
Respiratory symptoms	Cough, dyspnoea, sore throat, chest discomfort, nasal symptoms	Supports ARI characterization
Symptom duration and progression	Days since onset, worsening in last 24–48 h	Helps determine urgency and follow-up interval
Vital signs	Temperature, SpO_2_, respiratory rate when available, blood pressure when relevant	Supports deterioration detection
Comorbidities	Hypertension, diabetes, chronic lung disease, cardiovascular disease, immunosuppression	Refines risk stratification
Functional status	Confusion, weakness, reduced oral intake, inability to perform usual activities	Detects atypical deterioration in older adults
Caregiver observations	Narrative description, perceived severity, barriers to transport	Adds contextual information for decision-making
Digital access	Phone availability, connectivity, preferred communication channel	Determines feasibility of remote follow-up

**Table 3:** Illustrative risk-based teletriage logic for respiratory infection monitoring, with the explicit numerical thresholds implemented in the scenario-based evaluation (Section 2.8).

Risk Level	Indicative Criteria	Recommended Action
Low risk	Mild symptoms, no dyspnoea, stable general condition, normal or unavailable SpO_2_ (≥95% or unavailable, with no other warning sign)	Preventive education, home care guidance, remote follow-up in 24–48 h
Moderate risk	Persistent fever (≥2 days), comorbidities present, caregiver concern ≥4 on a 1–10 scale, mild dyspnoea, or borderline SpO_2_ (92–94%)	Priority teleconsultation, structured follow-up, clinician review of dashboard data
High risk	SpO_2_ <92%, severe dyspnoea, confusion, chest pain, respiratory rate ≥28 breaths/min, or caregiver concern ≥8 on a 1–10 scale	Immediate clinical escalation, urgent teleconsultation, emergency referral or in-person assessment

Note: The numerical thresholds presented in this table are illustrative and were derived from published clinical guidelines and consensus criteria for acute respiratory illness in older adults. They have not been formally validated in a prospective clinical study specific to this population, nor have they received formal sign-off from an institutional clinical panel for the present study. Prior to deployment in any real-world triage workflow, these thresholds must be subject to formal clinical review, local protocol adaptation, and prospective pilot validation.

**Table 4:** Conversion of empirical results into smart tele-primary-care design requirements.

Empirical Result	Public Health/Healthcare Clinical Interpretation	Digital Design Requirement
Good declarative knowledge but poor adherence	Good information does not guarantee sustained prevention	Individualized educational reinforcement and follow-up reminders
Poor bio-security practices	Routines involving older adults influence respiratory infection prevention	Contextual guidance based on caregiver capacity
Variable distancing or avoidance of physical greeting when upper respiratory symptoms are present	Perceived risk may be overridden by social norms	Messages to support behavior change and risk communication
Dependence upon caregivers among older adults	Caregivers mediate access to healthcare providers for observation and implementation of recommendations	Interface designed with focus on caregivers and structured symptom reporting
Contextual rural/low income setting	Potential barriers to healthcare access delay care seeking for acute conditions	Easy entry point and remote triage
Need for continuity between appointments	Single Consultations do not provide sufficient opportunity for monitoring of acute respiratory infections	Remote follow-up and dashboard-based case tracking

**Table 5:** Synthesized components of the proposed telehealth pathway for primary care.

Component	Primary Aim	Operational Focus
Access and triage	Facilitate entry into care	Identification of need, initial orientation, and routing according to urgency and feasibility of remote management
Synchronous teleconsultation	Provide timely clinical guidance	Virtual assessment, counseling, clarification of symptoms, and caregiver-inclusive decision support
Remote follow-up	Promote continuity of care	Monitoring of progress, reinforcement of recommendations, and identification of warning signs
Preventive education	Improve self-care and adherence	Culturally adapted education on hygiene, symptom recognition, respiratory precautions, and caregiver support

**Table 6:** Functional role of the LLM layer in the proposed tele-primary-care model.

LLM Component	Function	Input	Output	Safeguard
Conversational assistant	Collect symptoms from caregivers	Natural language	Structured symptom report	Human review
Educational module	Explain preventive measures	Caregiver questions	Tailored guidance	Validated content
Warning-sign detector	Identify predefined risk expressions	Symptoms and vital signs	Risk flag	Rule-based protocol
Summary generator	Support clinical workflow	Conversation history	Clinician-facing summary	Professional validation
Follow-up module	Maintain continuity	Patient status	Reminders and check-ins	Escalation rules

**Table 7:** Indicators proposed for a dashboard for primary-care teams.

Indicator	Description	Role in Care Management
Category of Risk	Low risk, moderate risk or high risk (based on protocol)	Defines priority for clinical review
Trajectory of Symptoms	Stabilized, improved or worsened symptoms	Defines level of follow-up needed
Trend SpO_2_	Available SpO_2_ readings	Identifies potential acute respiratory decline
Persistence of Fever	Length and frequency of fever	Defines appropriate time for telemedicine consult
Score for Concern of Caregiver	Standard scale of caregiver subjective estimate of illness severity	Catches contextual risk signals
Status of Follow-Up	Complete, pending, missed, escalated	Assists in ensuring continuity and accountability
Needs Education	Identified topics caregivers wish to learn about/identified misunderstandings by caregiver	Assists in providing focused education
History of Escalations	Number of alerts generated and action taken	Provides audit trail and opportunities for quality improvement

**Table 8:** Safeguarding measures for safe implementation of the LLM-mediated framework.

Risk	Potential Consequence	Strategy to Mitigate
Hallucinations or inappropriate recommendations due to incorrect AI guidance	Patient receives incorrect advice potentially resulting in patient injury/damage.	LLM utilizes validated knowledge-base; uses restricted prompts; has clinician review
Over-reliance on automated recommendations (automation bias)	Clinicians/Caregivers rely too heavily on AI’s output without appropriately validating its correctness.	Displays clear disclaimer statements regarding limitations of technology; requires manual validation by clinician/human
Failure to identify warning signs due to missed alert(s)	Delays referral to urgent/emergency department.	Establishes conservative alert rules/scripts; establishes emergency scripts
Data Privacy Breach	Health-related information/data of patients are exposed.	Minimize amount of data collected; encrypt data (e.g., end-to-end encryption between caregiver interface and dashboard); establish access controls and auditing logs
Disproportionate access to digital technologies (digital divide)	Individuals who do not possess sufficient literacy skills/or lack connectivity/access experience barriers in accessing technology.	Utilize alternative means such as mobile phone; provides caregiver-assisted technology use
Biases in Communication Patterns (e.g., language)	Healthcare communications fail to effectively communicate with individuals living in rural areas or those possessing low-literacy.	Develop local language adaptations; perform testing; continuously monitor
Accountability Gaps for Clinical Decision-Making	No clear definition exists regarding responsibilities/authority for making decisions.	Establish definitions for authority of clinician and define institutional governance policies/procedures

**Table 9:** Preliminary scenario-based assessment indicators for the proposed workflow.

Indicator	Simulated Value	Interpretation
Overall triage agreement	93.3%	High internal agreement between reference labels and workflow output (56/60 cases)
High-risk sensitivity	100.0%	All twenty high-risk scenarios were correctly escalated
High-risk specificity	92.5%	Three of forty non-high-risk cases were escalated as high risk, all via the missing-data safeguard
Balanced accuracy	93.8%	Stable preliminary performance across risk categories
Cohen’s kappa	0.899	Strong agreement beyond chance in the simulated dataset
Dataset completeness	85.0%	A pulse-oximetry reading was available for 51 of 60 scenarios; the remainder triggered the conservative missing-data rule

## Data Availability

The survey data analyzed in this study are available from the corresponding author upon reasonable request, in accordance with national data-protection regulations and the informed-consent terms under which they were collected; only de-identified, aggregated summary data are reported in this manuscript. The reproducible Python/Colab workflow used to generate the figures and the scenario-based assessment reported in this study—including the deterministic scenario-construction code, the resulting scenario_inputs.csv file, the rule-based teletriage function, and the simulated_respiratory_validation_cases.csv and simulated_validation_metrics.csv files it produces—is available at https://github.com/yairriverajul/LLM (accessed on 3 July 2026). The code itself contains no patient data.
